# Left Atrial Spontaneous Echo Contrast and Ischemic Stroke in Patients Undergoing Percutaneous Left Atrial Appendage Closure

**DOI:** 10.3389/fcvm.2021.723280

**Published:** 2021-09-23

**Authors:** Binhao Wang, Zhao Wang, Guohua Fu, Bin He, Hangxuan Wang, Weidong Zhuo, Shengmin Zhang, Huimin Chu

**Affiliations:** ^1^Arrhythmia Center, Ningbo First Hospital, Ningbo, China; ^2^Department of Ultrasonography, Ningbo First Hospital, Ningbo, China

**Keywords:** atrial fibrillation, percutaneous left atrial appendage closure, spontaneous echo contrast, device-related thrombus, stroke

## Abstract

**Objectives:** Spontaneous echo contrast (SEC) in the left atrium (LA) is frequently observed in atrial fibrillation (AF) patients and may lead to thromboembolic events. We aimed to investigate both periprocedural and long-term stroke risks associated with LA SEC in AF patients undergoing percutaneous left atrial appendage closure (LAAC).

**Methods:** A total of 408 consecutive AF patients treated with LAAC between March 2015 and February 2019 were divided into two groups based on preprocedural transesophageal echocardiography: the study group (moderate/severe LA SEC; *n* = 41) and the control group (none, mild, or mild to moderate LA SEC; *n* = 367). To attenuate the observed imbalance in baseline covariates, a propensity score matching technique was used.

**Results:** No periprocedural stroke/transient ischemic attack (TIA) was documented. The incidence of device-related thrombus was higher in the study group than in the control group (8.8 vs. 1.3%; *P* = 0.025). The mean follow-up period was 3.2 ± 1.1 years, during which 8 patients (2.2%) in the control group and 4 (9.8%) in the study group experienced stroke/TIA (*P* = 0.024). Moderate/severe LA SEC was identified as an independent predictor of stroke/TIA in both the original population (HR = 5.71, 95% CI 1.47–22.19, *P* = 0.012) and the matched population (HR = 9.79, 95% CI 1.44–66.86, *P* = 0.020).

**Conclusions:** LA SEC did not show a relationship with periprocedural stroke events in patients undergoing percutaneous LAAC. However, moderate/severe LA SEC increased the incidence of device-related thrombus and the risk of late stroke/TIA.

## Introduction

Atrial fibrillation (AF) is the most common arrhythmia in the clinical setting (1). Ischemic stroke is one of the most serious complications of AF (2). The majority of thrombi originate from the left atrial appendage (LAA) (3). The anticoagulation treatment strategy is typically chosen based on the CHA_2_DS_2_-VASc score in patients with AF. However, no echocardiographic parameter is considered for stroke risk stratification (4). Spontaneous echo contrast (SEC) in the left atrium (LA) is frequently observed on transesophageal echocardiography (TEE) in AF patients. Recently, Gedikli et al. reported that the presence of dense LA SEC did not show any correlation with periprocedural thromboembolic events in AF patients undergoing catheter ablation with uninterrupted anticoagulation. However, a significant association was observed with late stroke/transient ischemic attack (TIA) (5).

Percutaneous LAA closure (LAAC) is a kind of non-pharmacologic alternative for stroke prevention in patients with AF (6). Sedaghat et al. reported that patients with DRT after LAAC had higher SEC grades within the LAA (7). However, the impact of LA SEC on stroke risk in patients after LAAC is unclear. Therefore, we investigated both periprocedural and long-term stroke risk associated with LA SEC in AF patients undergoing percutaneous LAAC in our center.

## Materials and Methods

### Study Population

We performed a single-center prospective cohort study of consecutive AF patients undergoing percutaneous LAAC at our center from March 2015 to February 2019 who also had undergone pre-procedural TEE. All clinical, echocardiographic, and periprocedural baseline data were prospectively collected. The CHA_2_DS_2_-VASc score and HAS-BLED score were calculated by two investigators for each patient based on comorbidities.

Cervical vascular ultrasound examination was performed with standardized technology. Extracranial carotid arteries were screened for arterial intima-media thickness (IMT), plaques, and stenosis. IMT ≥ 1.0 mm was considered thickened; IMT ≥ 1.5 mm was defined as plaque. The degree of carotid artery stenosis was divided into non-stenosis, 1–49% (mild), 50–69% (moderate), 70–99% (severe), and occlusive (8).

The inclusion criteria were as follows: age > 18 years; non-valvular AF; CHA_2_DS_2_-VASc score ≥ 2; HAS-BLED score ≥ 3; contraindications for long-term oral anticoagulation (OAC) therapy (e.g., patients with previous bleeding events or thromboembolic events under OAC or intolerance); or refusal to take OAC medication. The exclusion criteria were thrombus formation in the LA; AF in the setting of moderate-to-severe mitral stenosis and/or in the presence of a mechanical heart valve; acute myocardial infarction or unstable angina; prior stroke or transient attack within 30 days; acute infective endocarditis; uncontrolled hemorrhagic disease; pregnancy; and the presence of an atrial septal repair or a closure history.

This study was conducted in compliance with the law protecting personal data in accordance with the guidelines of the Helsinki Declaration. The study was approved by the Ethics Committee of Ningbo First Hospital, and written informed consent for the percutaneous LAAC procedure was obtained from all patients.

### TEE Examination

TEE was performed 24 h preprocedurally using a PHILIPS EPIQ7C device (PHILIPS, Amsterdam, Netherlands). The gain was continuously adjusted until acquisition of the best image. The presence or absence of LA SEC was determined by 2 experienced echocardiographers. LA SEC was defined as an echogenic, swirling pattern of blood flow at the standard gain setting during the cardiac cycle and was graded according to Fatkin classification (9).

### Percutaneous LAAC

All procedures were performed with the patient under uninterrupted anticoagulation. LAAC devices, including Watchman (Boston Scientific, Natick, Massachusetts), the Amplatzer Cardiac Plug (ACP, St. Jude Medical, Saint Paul, Minnesota), and LAmbre (Lifetech Scientific, Shenzhen, China) were selected by the operator according to the pre-procedural TEE measurements and LAA angiogram results. The methods used for device implantation have been published previously (6, 10, 11). Briefly, all procedures were performed by an experienced operator in our center (HMC). After transseptal puncture, the transseptal sheath was exchanged with a delivery sheath, and intravenous heparin was administered to achieve an activated clotting time of more than 250 s. The device was advanced into the LAA through the delivery sheath and deployed via sheath retraction. A TEE probe was placed to assess the position of the device. A successful device position was defined as no or minimal contrast peridevice leakage ≤ 5 mm into the LAA according to TEE. A gentle tug test was performed to ensure device stability. The device was released after confirmation of an adequate position and a tug test. The grades of LA SEC were re-evaluated after the device was released.

Transthoracic echocardiography was performed on day 1 post-procedure to exclude device embolization and pericardial effusion. Thromboembolic events included ischemic stroke, TIA and systemic embolism. Bleeding events were classified as major (intracranial, retroperitoneal, intraspinal, intraocular or pericardial hemorrhage; decrease in hemoglobin > 2 g/dL; transfusion of ≥2 units of packed red blood cells) or minor (remaining types of bleeding events) (12). The major periprocedural adverse events included death, stroke, TIA, device embolization and major bleeding events. The minor complications included minor bleeding or vascular complications (e.g., arteriovenous fistula, femoral hematoma, and pseudoaneurysm) without the need for further intervention.

### Follow-Up

All of the patients were followed until August 31, 2020. The antithrombotic medication strategy was chosen by the operators based on the stroke and bleeding risk. For the first 6 weeks after the procedure, most patients were treated with OACs [warfarin with an international normalized ratio (INR) of 2–3, 15–20 mg rivaroxaban once daily, or 110–150 mg dabigatran twice daily] followed by dual antiplatelet therapy (DAPT, 100 mg aspirin and 75 mg clopidogrel once daily) between 6 weeks and 6 months and single antiplatelet therapy (SAPT, 100 mg aspirin or 75 mg clopidogrel once daily) after 6 months. Patients with contraindications for OACs and those who refused to take OACs were treated with DAPT for the first 6 months, followed by SAPT. Clinical follow-up was arranged at 6 weeks, 3 months, 6 months, and 12 months post-procedure. TEE follow-up was scheduled approximately 6 weeks, 6 months, and 12 months after the procedure to evaluate the device position, peridevice leakage, and DRT. Computed tomography (CT) was the alternative choice if the patient refused to undergo TEE.

### Clinical Outcomes

Clinical outcomes included death, ischemic stroke, TIA, systemic embolism, and major bleeding. All patients were monitored for thromboembolic complications by follow-up TEE, clinic visits, and phone calls by our research staff. Stroke was defined as the onset of a new neurologic deficit that occurred any time after LAAC and persisted for >24 h. It was confirmed by cerebral magnetic resonance imaging or CT and determined by at least two radiologists or neurologists. If the duration of the deficit was <24 h, it was defined as a TIA. A TIA/stroke occurring within 48 h of the LAAC procedure was considered a periprocedural event.

Device efficacy for preventing stroke and reducing major bleeding events was tested by comparing the actual event rate during follow-up with the predicted event rate by the CHA_2_DS_2_-VASc score and HAS-BLED score. The individual patient annual risk was recorded, and the average annual risk was calculated. The total number of thromboembolic events during both the periprocedural and follow-up periods was divided by the total patient-years of follow-up and multiplied by 100 to obtain the actual annual rate. Thromboembolism and bleeding reduction were calculated as follows: (estimated – actual event rate)/estimated event rate.

### Statistical Analysis

Based on the TEE findings, patients were divided into 2 groups: patients with moderate/severe LA SEC (study group) and patients with none, mild or mild to moderate LA SEC (control group). Normally distributed continuous variables are expressed as the mean (standard deviation), while the median (interquartile range) is used for variables with a skewed distribution. Categorical variables are expressed as absolute numbers (percentages). Continuous variables were compared using the *t*-test and Mann-Whitney U test for normally and non-normally distributed data, respectively. Categorical variables were compared using the chi-square test or Fisher's exact test where appropriate.

Survivor functions were estimated for each group using the Kaplan-Meier method and statistically evaluated using a log-rank test of trend. We used the Cox proportional hazards model with the control group as the reference to calculate unadjusted and adjusted hazard ratios (HRs) and corresponding 95% confidence intervals (CIs) for the occurrence of stroke/TIA. The initial model was unadjusted, and the subsequent model was adjusted for age, sex, and body mass index. The multivariable adjusted model included age, sex, body mass index, AF type, CHA_2_DS_2_-VASc score, LA diameter, peridevice leakage, type of LAAC device, DRT, and type of antithrombotic medication post-procedure (time-varying covariate as the regimen changed over time). Statistical analyses were performed with SPSS 19.0 (IBM, Armonk, NY, USA), and *P* < 0.05 (2-tailed) was considered to be statistically significant.

To attenuate the observed imbalance in baseline covariates between the study groups, a propensity score matching technique was used. Propensity scores were computed conditional on the probability of moderate/severe LA SEC detection at baseline TEE by fitting a multivariable logistic regression model (covariates were age, sex, AF type, pre-procedure antithrombotic regimen, carotid atherosclerosis, LA diameter and CHA_2_DS_2_-VASc score). The caliper matching technique with a 0.02 propensity score tolerance on the maximum propensity score distance (caliper width) was used in the matching algorithm. A one-to-two pair matching method with a specified tolerance distance was used to identify matched cohorts. This method resulted in a propensity score-matched population with 41 and 123 patients in the study group and control group, respectively.

## Results

### Baseline Characteristics

**Figure 1:**
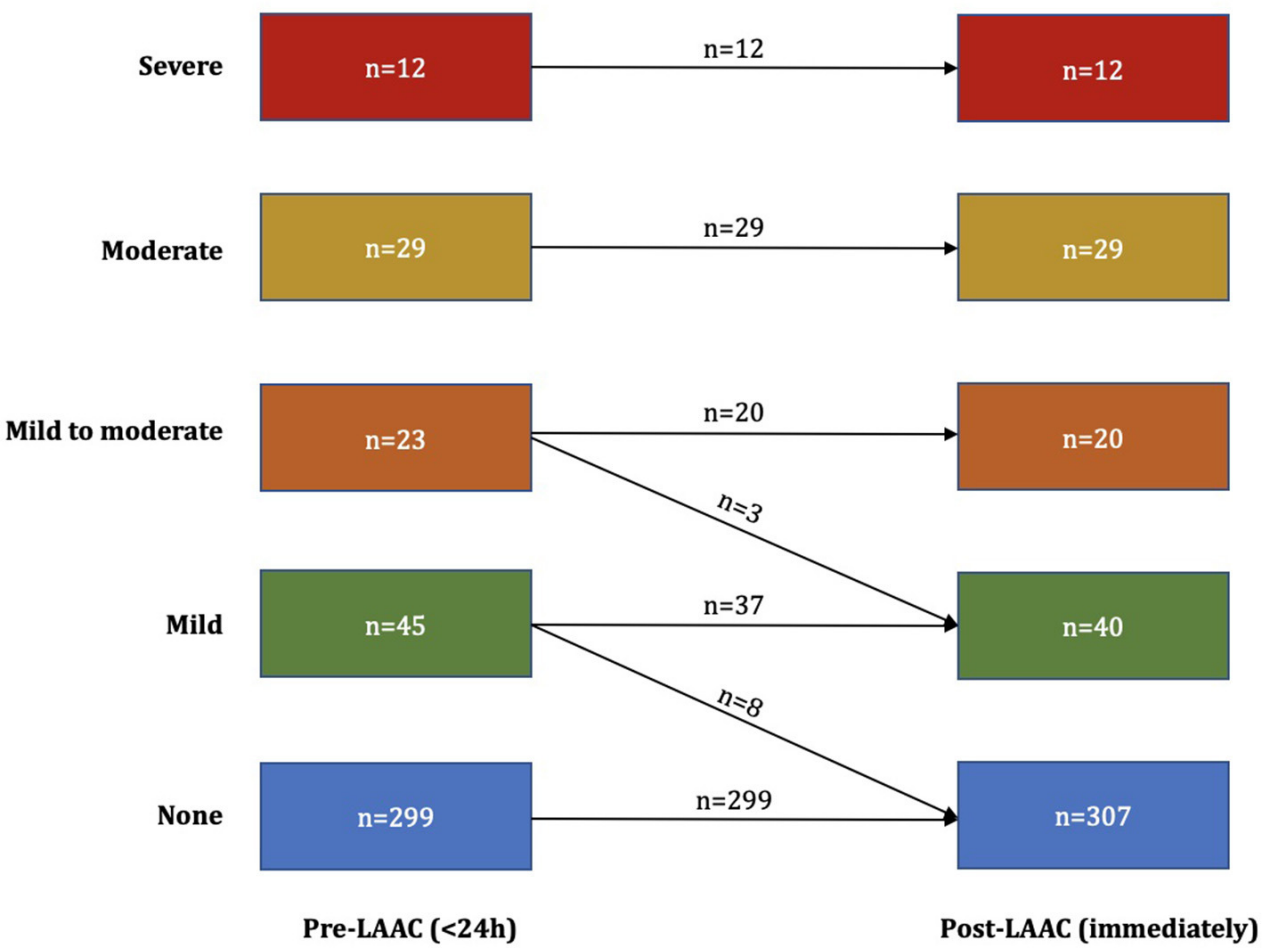
Left atrial spontaneous echo contrast grades before and after left atrial appendage closure (LAAC).

**Figure 2:**
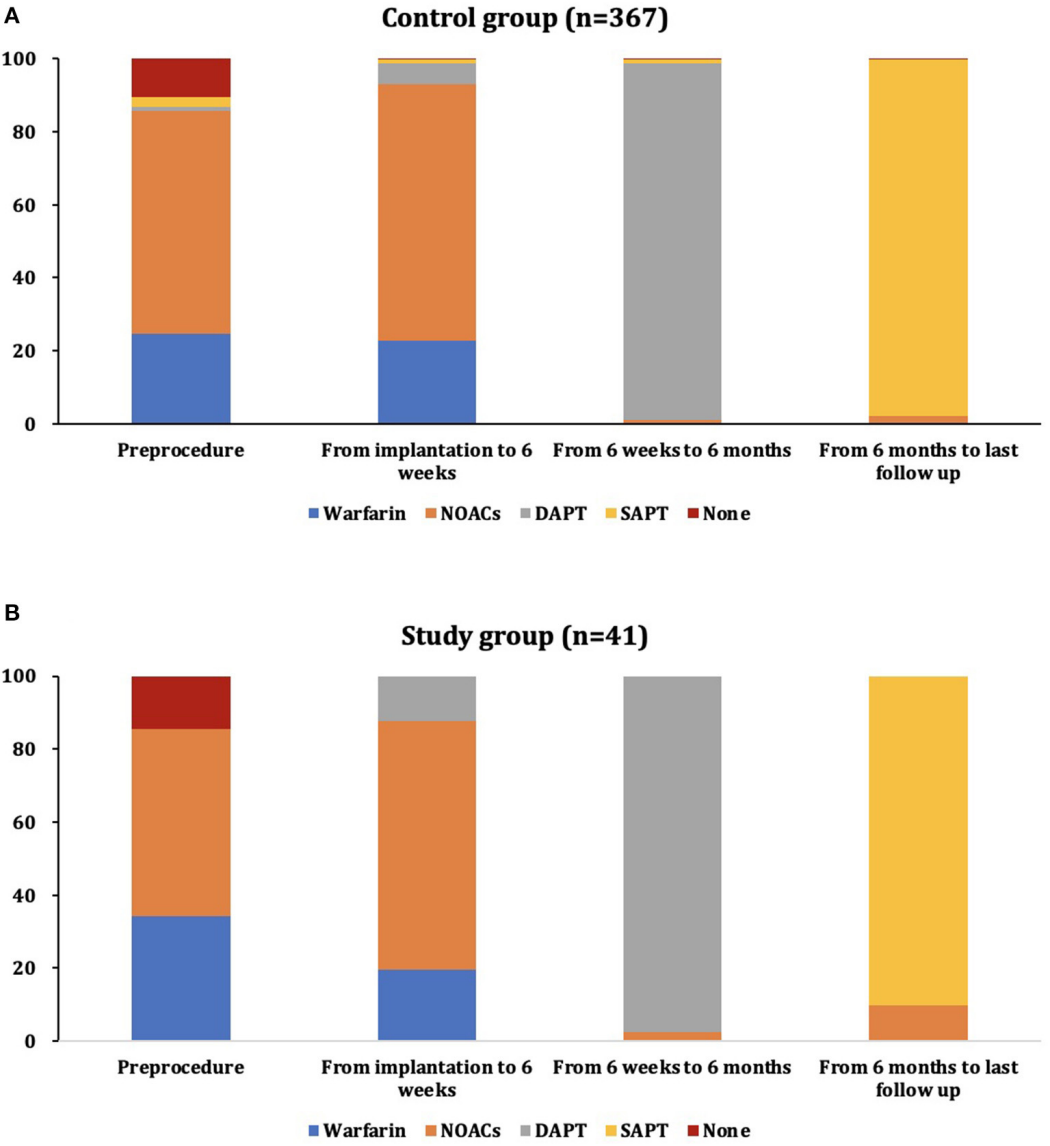
The distribution of antithrombotic therapy during different periods of follow-up in the control group **(A)** and study group **(B)**. DAPT, dual antiplatelet therapy; NOAC, non-vitamin K antagonist oral anticoagulant; SAPT, single antiplatelet therapy.

A total of 408 patients with AF undergoing LAAC at our center who also had undergone pre-procedural TEE from March 2015 to February 2019 were included in this analysis. Moderate/severe LA SEC was detected in 41 patients (10.0%) on TEE performed at baseline (study group); the control group (none, mild, or mild to moderate LA SEC on baseline TEE) included 367 patients (Figure 1). Baseline characteristics are displayed in Table 1. The percentage of paroxysmal AF was greater in the control group (34.3 vs. 14.6%, *P* = 0.026). The CHA_2_DS_2_-VASc score and HAS-BLED score were comparable between the groups. The LAA orifice diameter was larger in the patients implanted with nitinol plug devices (ACP or LAmbre) than in those implanted with nitinol cage devices (Watchman) (Supplementary Table 1). There was no difference in antithrombotic medication pre-procedurally; more than half of the patients were taking non-vitamin K antagonist oral anticoagulants (NOACs) (Figure 2). Among the patients taking warfarin, INR in 92.3% (84/91) and 57.1% (8/14) was in the therapeutic range in the control group and study group, respectively.

**Table 1:** Baseline characteristics of the study population.

	**Unmatched population**			**Propensity score-matched population**		
	**Control group (*n* = 367)**	**Study group (*n* = 41)**	***P*-value**	**Control group (*n* = 123)**	**Study group (*n* = 41)**	***P*-value**
Age, years	69.5 ± 8.7	69.6 ± 9.5	0.952	69.5 ± 8.0	69.6 ± 9.5	0.983
Male	253 (64.0)	27 (65.9)	0.818	84 (68.3)	27 (65.9)	0.772
Body mass index, kg/m^2^	24.4 ± 3.4	24.4 ± 3.6	0.916	24.5 ± 3.6	24.4 ± 3.6	0.709
Paroxysmal AF	126 (34.3)	6 (14.6)	0.026	19 (15.4)	6 (14.6)	0.900
Hypertension	244 (66.5)	30 (73.2)	0.387	83 (67.5)	30 (73.2)	0.495
Coronary artery disease	34 (9.3)	4 (9.8)	0.784	10 (8.1)	4 (9.8)	0.751
Diabetes	68 (18.5)	3 (7.3)	0.073	9 (7.3)	3 (7.3)	1.000
Congestive heart failure	44 (12.0)	7 (17.1)	0.351	19 (15.4)	7 (17.1)	0.805
Carotid atherosclerosis			0.412			0.739
IMT thickening	56 (15.3)	9 (22.0)		20 (16.3)	9 (22.0)	
Plaque formation	198 (54.0)	23 (56.1)		74 (60.2)	23 (56.1)	
Mild stenosis	7 (1.9)	1 (2.4)		2 (1.6)	1 (2.4)	
Previous stroke/TIA	270 (73.6)	28 (68.3)	0.470	83 (67.5)	28 (68.3)	0.923
Previous bleeding	97 (26.4)	11 (26.8)	0.956	35 (28.5)	11 (26.8)	0.841
CHA_2_DS_2_-VASc score	4.6 ± 1.5	4.4 ± 1.7	0.448	4.4 ± 1.5	4.4 ± 1.7	0.748
HAS-BLED score	3.1 ± 0.9	3.1 ± 0.8	0.939	3.2 ± 1.0	3.1 ± 0.8	0.640
LA diameter, mm	44.5 ± 7.3	48.7 ± 6.6	0.001	48.4 ± 7.3	48.7 ± 6.6	0.825
LVEF, %	61.9 ± 6.8	61.3 ± 5.7	0.567	61.8 ± 6.4	61.3 ± 5.7	0.680
LAA orifice diameter, mm	24.7 ± 4.9	25.3 ± 4.6	0.476	26.0 ± 5.4	25.3 ± 4.6	0.452
LAA depth, mm	29.4 ± 5.8	31.0 ± 4.8	0.081	30.3 ± 6.0	31.0 ± 4.8	0.083

*AF, atrial fibrillation; LA, left atrial; LAA, left atrial appendage; LVEF, left ventricular ejection fraction; TIA, transient ischemic attack*.

Because of differences in the key baseline characteristics, we used the propensity score matching method. In the 1:3 propensity score matched dataset, all variables were well-balanced between the study group (*n* = 41) and the control group (*n* = 123) (Table 1).

### Periprocedural Data

As shown in Table 2, the device was successfully implanted in all patients. Thirty-five (9.5%) and 4 (9.8%) patients had peridevice leakages <5 mm. The grades of LA SEC immediately after LAAC are displayed in Figure 1. Patients with moderate/severe LA SEC before LAAC maintained the original grades. One patient in the control group experienced device embolization. His LAA was occluded by LAmbre 3036. He complained of chest pain on the evening of the procedure day and was confirmed to have device embolization into the left ventricle by echo examination. The percutaneous technique failed, and surgical retrieval was performed. Five (1.4%) patients in the control group experienced cardiac tamponade and were treated with pericardiocentesis. Two (0.5%) patients in the control group and 1 (2.4%) in the study group had pericardial effusion post-procedure. They all recovered without any treatment. No patients experienced death or stroke.

**Table 2:** Peri-procedural data.

	**Unmatched population**			**Propensity score-matched population**		
	**Control group (*n* = 367)**	**Study group (*n* = 41)**	***P*-value**	**Control group (*n* = 123)**	**Study group (*n* = 41)**	***P*-value**
LAA closure device			0.418			0.383
Watchman	230 (62.7)	23 (56.1)		77 (62.6)	23 (56.1)	
Amplatzer Cardiac Plug	91 (24.8)	14 (34.1)		29 (23.6)	14 (34.1)	
LAmbre	46 (12.5)	4 (9.8)		17 (13.8)	4 (9.8)	
LAA leakage			1.000			1.000
>5 mm	0	0		0	0	
≤5 mm	35 (9.5)	4 (9.8)		12 (9.6)	4 (9.8)	
Complications						
Death	0	0	–	0	0	–
Stroke/TIA	0	0	–	0	0	–
Device embolization	1 (0.3)	0	1.000	1 (0.3)	0	1.000
Pericardial tamponade	5 (1.4)	0	1.000	2 (1.6)	0	1.000
Pericardial effusion	2 (0.5)	1 (2.4)	0.273	2 (1.6)	1 (2.4)	1.000
Minor complications^†^	12 (3.3)	2 (4.9)	0.641	5 (4.1)	2 (4.9)	1.000

† *Including minor bleeding, arteriovenous fistula, femoral hematoma, and pseudoaneurysm*.

*LAA, left atrial appendage; TIA, transient ischemic attack*.

### Follow-Up Data

Figure 2 shows the antithrombotic medication strategy used during follow-up. The type of OAC was switched to NOACs in patients taking warfarin and without a therapeutic range of INR (7 in the control group and 6 in the study group). A total of 301 (82.0%; 293 TEE, 8 CT) and 34 (82.9%; 32 TEE, 2 CT) patients received TEE or CT at least once during the follow-up period in the control group and study group, respectively (Table 3). Most patients taking warfarin were in the therapeutic range (97.6% in the control group and 100% in the study group). DRT was detected in 4 (1.3%) patients in the control group and 3 (8.8%) in the study group (*P* = 0.025) (see details in Table 4). The incidence of DRT was comparable between nitinol cage and plug devices (Supplementary Table 2). The incidence of DRT was higher in patients taking DAPT after LAAC than in those taking OACs (4.0% vs. 1.9%). However, the difference did not reach statistical significance (*P* = 0.422) (Supplementary Table 3). DRT was detected in 3 patients by TEE 6 weeks after the LAAC procedure when taking warfarin with the therapeutic INR. DRTs were detected by TEE 6 months after the LAAC procedure in 2 patients taking DAPT. The remaining 2 patients presented DRT detected by TEE 12 months after the LAAC procedure when taking SAPT. All patients with DRT received NOACs until the thrombus dissolved. None of the patients with DRTs experienced stroke/TIA during follow-up. No peridevice leakage > 5 mm was detected in either group.

**Table 3:** Follow-up data.

	**Unmatched population**			**Propensity score-matched population**		
	**Control group (*n* = 367)**	**Study group (*n* = 41)**	***P*-value**	**Control group (*n* = 123)**	**Study group (*n* = 41)**	***P*-value**
Antithrombotic medication			0.222			0.776
OACs	341 (92.9)	36 (87.8)		110 (89.4)	36 (87.8)	
DAPT	26 (7.1)	5 (12.2)		13 (10.6)	5 (12.2)	
**Adverse events**
Death	1 (0.3)	0	1.000	0	0	–
Stroke/TIA	8 (2.2)	4 (9.8)	0.024	2 (1.6)	4 (9.8)	0.035
Stroke	7 (1.9)	4 (9.8)	0.017	2 (1.6)	4 (9.8)	0.035
TIA	1 (0.3)	0	1.000	0	0	–
Cerebral hemorrhage	1 (0.3)	0	1.000	0	0	–
Gastrointestinal bleeding	1 (0.3)	0	1.000	0	0	–
TEE or CT follow-up			0.475			0.600
TEE	293 (79.8)	32 (78.0)		98 (79.7)	32 (78.0)	
CT	8 (2.2)	2 (4.9)		3 (2.3)	2 (4.9)	
Imagine follow-up results						
DRT in patients with TEE/CT	4 (1.3)	3 (8.8)	0.025	1 (1.0)	3 (8.8)	0.049
Residual flow > 5 mm	0	0	1.000	0	0	1.000
Residual flow ≤ 5 mm	29 (8.8)	3 (8.8)		9 (8.9)	3 (8.8)	

*DAPT, Dual antiplatelet therapy; DRT, device-related thrombus; CT, computed tomography; OAC, oral anticoagulation; TIA, transient ischemic attack; TEE, transesophageal echocardiography*.

**Table 4:** DRT and clinical outcomes.

**Events**	**Age**	**Sex**	**Device type**	**Days after LAAC**	**Rhythm status**	**Degree of LA SEC**	**Antithrombotic medication**
**Study group**
DRT	70	Male	Watchman	42	AF	Moderate	Warfarin
DRT	63	Female	Watchman	176	Sinus rhythm	Moderate	DAPT
DRT	59	Female	LAmbre	360	AF	Severe	Aspirin
Stroke	74	Male	Watchman	1,238	Sinus rhythm	Moderate	Aspirin
Stroke	70	Male	ACP	1,247	AF	Severe	Aspirin
Stroke	78	Female	Watchman	961	AF	Severe	Clopidogrel
Stroke	69	Male	ACP	105	AF	Moderate	DAPT
**Control group**
DRT	75	Male	ACP	45	Sinus rhythm	None	Warfarin
DRT	71	Female	Watchman	43	AF	None	Warfarin
DRT	68	Male	ACP	184	AF	Mild	DAPT
DRT	70	Male	Watchman	368	AF	None	Clopidogrel
TIA	61	Male	Watchman	511	AF	None	Aspirin
Stroke	68	Male	ACP	12	AF	None	Dabigatran
Stroke	70	Male	Watchman	44	Sinus rhythm	None	Dabigatran
Stroke	63	Male	ACP	520	AF	Mild	Aspirin
Stroke	82	Female	ACP	145	AF	None	DAPT
Stroke	63	Female	Watchman	623	AF	Mild	Clopidogrel
Stroke	69	Male	Watchman	124	AF	None	DAPT
Stroke	83	Male	Watchman	134	Sinus rhythm	Mild	DAPT
Death	82	Female	ACP	145	Sinus rhythm	Unknown	DAPT

*AF, atrial fibrillation; DAPT, Dual antiplatelet therapy; DRT, device-related thrombus; LAAC, left atrial appendage closure; LA SEC, left atrial spontaneous echo contrast*.

**Figure 3:**
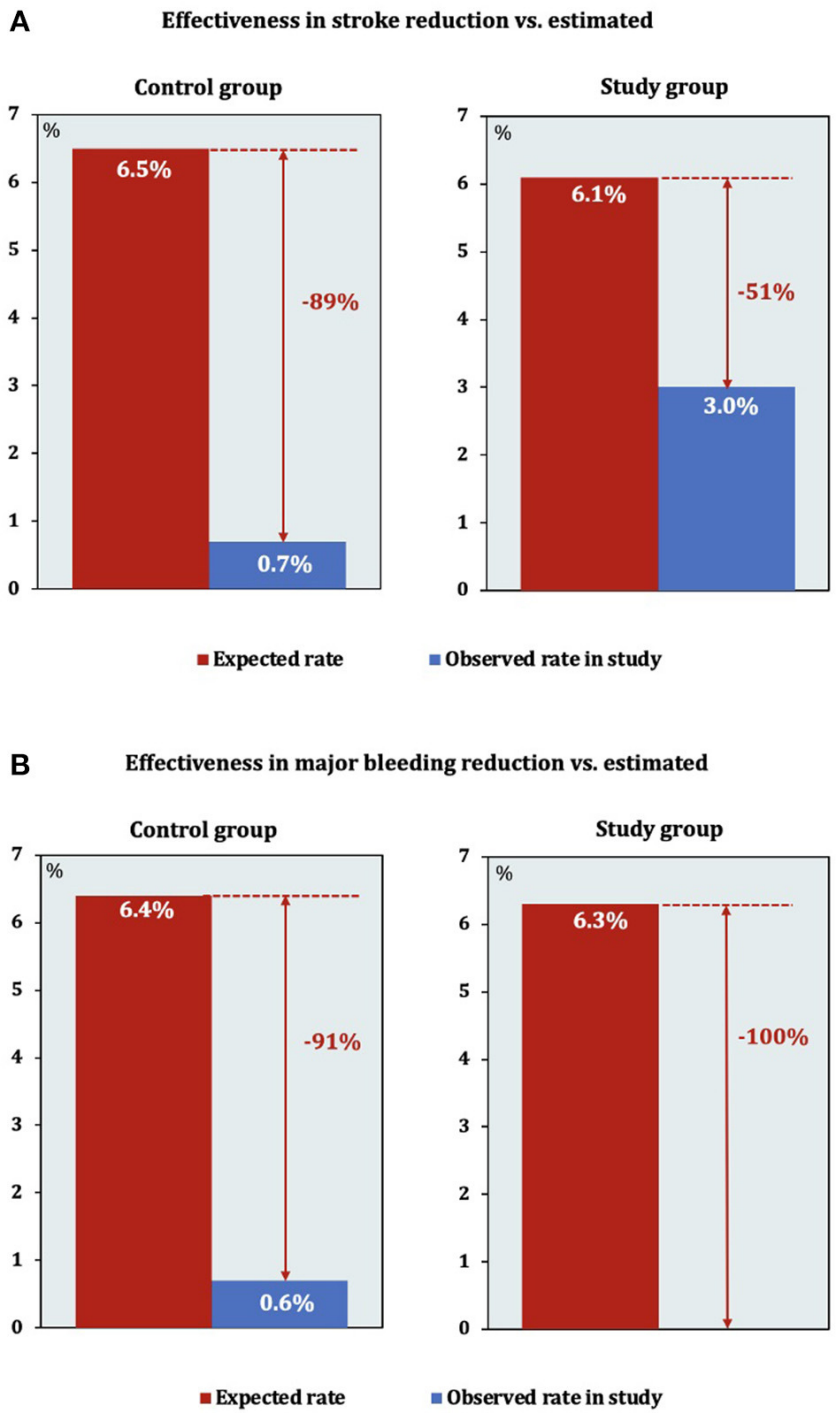
Effectiveness of left atrial appendage closure (LAAC) in reducing stroke and bleeding based on the annual rate predicted by the CHA_2_DS_2_-VASc score and HAS-BLED score in the control group **(A)** and study group **(B)**. Both periprocedural and follow-up events were included in the analysis.

Ischemic stroke/TIA occurred in 12 patients of the total study population [8 in the control group (7 stroke, 1 TIA), 4 in the study group (all stroke)] (see details in Table 4). Two patients in the control group and 1 in the study group had carotid plaque at baseline. DRT was not detected in any of the patients at the TEE follow-up. A total of 9 patients received intracranial CT angiography, and only 1 had plaque. All the patients were conservatively managed and recovered with only slight sequelae. The incidence of stroke/TIA remained higher in patients showing moderate/severe LA SEC, even after addressing the baseline imbalance through propensity score matching. In the matched population, 2 (1.6%) strokes occurred in the control group, and 4 (9.8%) occurred in the study group (*P* = 0.028). The incidence of stroke/TIA was comparable between patients taking DAPT and patients taking OACs after LAAC (3.2 vs. 2.9%) (Supplementary Table 3). A similar result was achieved between nitinol cage and plug devices (Supplementary Table 2). The annual ischemic stroke rate was 0.7/100 person-years, resulting in an 89% reduction compared with the expected annual risk in the control group, while the rate was 3.0/100 person-years, resulting in a 51% reduction in the study group (Figure 3).

One patient in the control group suffered a cerebral hemorrhage 5 months post-procedure and died. Gastrointestinal bleeding was documented in 1 patient in the control group. His condition improved with conservative treatment. No death or major bleeding events occurred during the follow-up in the study group. The annual major bleeding rate (including both periprocedural and follow-up events) was 0.6/100 person-years in the control group, which translated into a 91% reduction. The patients in the study group were free from major bleeding periprocedure and during follow-up (Figure 3).

### LA SEC and Stroke/TIA

**Figure 4:**
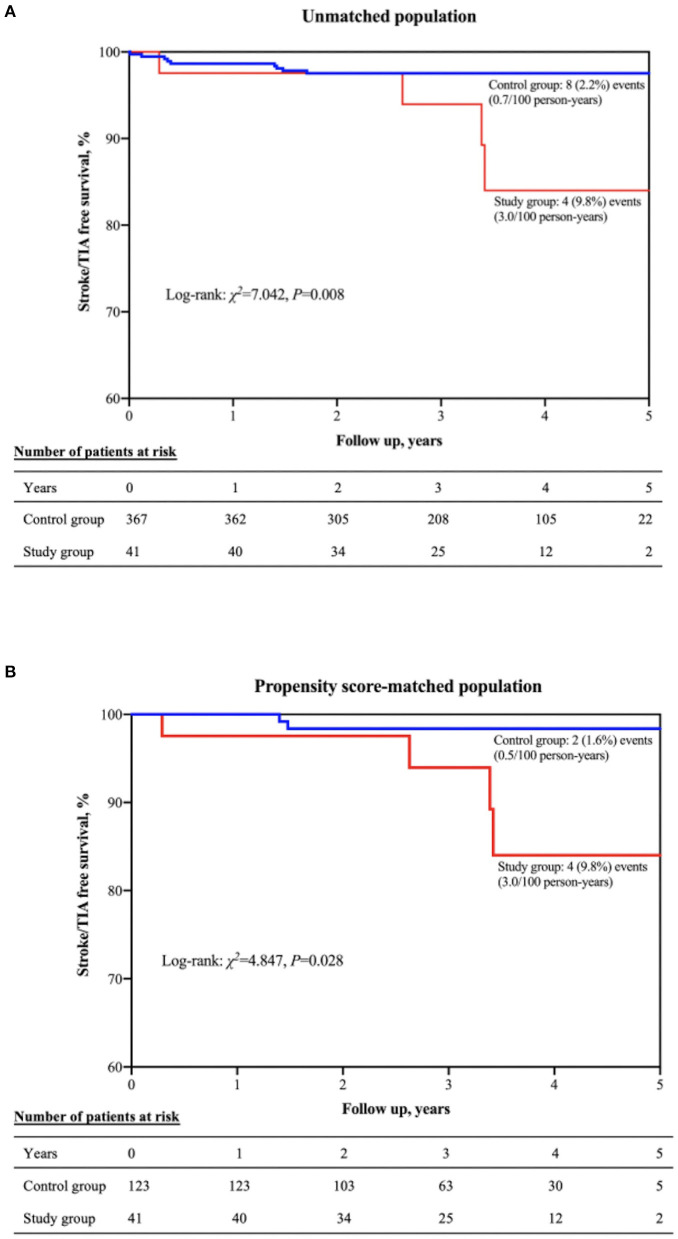
Kaplan-Meier curve showing stroke/TIA–free survival in the unmatched **(A)** and propensity score-matched **(B)** populations. TIA, transient ischemic attack.

After a mean follow-up period of 3.2 ± 1.1 years, 8 (0.7/100 person-years) patients in the control group and 4 (3.0/100 person-years) patients in the study group experienced stroke/TIA. Figure 4 displays the Kaplan-Meier survival curve for stroke/TIA according to the presence or absence of moderate/severe LA SEC. Moderate/severe LA SEC was associated with a higher risk of stroke/TIA during follow-up. After adjusting for all variables potentially associated with stroke, moderate/severe LA SEC was the only independent predictor of stroke/TIA (HR = 5.71, 95% CI 1.47–22.19, *P* = 0.012) and stroke alone (HR = 5.57, 95% CI 1.45–21.45, *P* = 0.013). The association remained significant in the matched population (HR = 9.79, 95% CI 1.44–66.86, *P* = 0.020) (Table 5).

**Table 5:** Univariable and multivariable analysis (Cox regression model) for predictors of stroke/TIA.

	**Univariable analysis**		**Multivariable analysis**	
	**HR (95% CI)**	***P*-value**	**HR (95% CI)**	***P*-value**
**Unmatched population**
Age, per 1 year increase	1.03 (0.96–1.10)	0.422	1.01 (0.93–1.09)	0.838
Body mass index, per 1 kg/m^2^ increase	0.94 (0.80–1.11)	0.449	0.96 (0.80–1.16)	0.680
Male	1.26 (0.39–4.10)	0.697	1.64 (0.45–6.01)	0.454
Non-paroxysmal AF	1.68 (0.46–6.10)	0.433	2.01 (0.47–8.71)	0.349
CHA_2_DS_2_-VASc score	1.16 (0.80–1.69)	0.439	1.22 (0.74–2.00)	0.445
LA diameter, per 1 mm increase	0.97 (0.90–1.05)	0.495	0.94 (0.85–1.04)	0.201
Moderate/severe LA SEC	3.93 (1.21–12.77)	0.023	5.71 (1.47–22.19)	0.012
Peridevice leakage	0.68 (0.18–2.47)	0.558	0.85 (0.22–3.26)	0.811
Nitinol plug device	0.95 (0.31–2.91)	0.929	1.02 (0.32–3.30)	0.682
Antithrombotic medication				
APT	Reference		Reference	
OAC	1.03 (0.13–7.94)	0.976	1.57 (0.18–13.49)	0.682
**Propensity score-matched population**
Age, per 1 year increase	1.04 (0.94–1.15)	0.475	1.05 (0.93–1.17)	0.457
Body mass index, per 1 kg/m^2^ increase	0.98 (0.78–1.24)	0.870	1.00 (0.76–1.33)	0.988
Male	2.34 (0.27–20.00)	0.439	2.88 (0.29–29.02)	0.370
Non-paroxysmal AF	0.85 (0.10–7.27)	0.881	0.61 (0.06–6.56)	0.680
CHA_2_DS_2_-VASc score	1.12 (0.65–1.94)	0.680	1.14 (0.54–2.41)	0.724
LA diameter, per 1 mm increase	0.98 (0.87–1.10)	0.718	0.96 (0.84–1.11)	0.607
Moderate/severe LA SEC	5.47 (1.00–29.97)	0.050	9.79 (1.44–66.86)	0.020
Peridevice leakage	1.18 (0.22–6.43)	0.851	2.05 (0.29–14.24)	0.469
Nitinol plug device	1.25 (0.23–6.84)	0.795	1.67 (0.24–11.74)	0.608
Antithrombotic medication				
APT	Reference		Reference	
OAC	0.61 (0.07–5.23)	0.651	0.97 (0.08–11.74)	0.608

*AF, atrial fibrillation; APT, antiplatelet therapy; CI, confidence interval; HR, hazard ratio; LA SEC, left atrial spontaneous echo contrast; OAC, oral anticoagulation; TIA, transient ischemic attack*.

## Discussion

### Main Findings

To our knowledge, this is the first prospective investigation of the predictive association of LA SEC with stroke risk in AF patients undergoing LAAC. The main findings of the present study were as follows: (1) no periprocedural stroke/TIA occurred in patients with moderate/severe LA SEC; (2) a significantly higher number of DRT and late stroke/TIA was reported in patients with moderate/severe LA SEC; and (3) the risk of stroke was 5.71 times higher in patients with moderate/severe LA SEC than in those without or with mild/mild to moderate LA SEC in the original population and 9.79 times higher in the propensity-matched population.

### SEC and Stroke

SEC is a sign of interaction between slowly moving red blood cells and plasma proteins (13) and is a marker of a hypercoagulable state (14). In our study population, moderate/severe LA SEC was detected in 41 of 408 patients (10.0%), which is similar to the findings in other investigations (5, 15). No stroke/TIA events occurred during the periprocedural period in patients with moderate/severe LA SEC in our study. Gedikli et al. also reported that no periprocedural thromboembolic events were observed in a dense smoke group undergoing AF ablation (5). Therefore, the presence of moderate/severe LA SEC did not increase the stroke risk in the periprocedural period.

However, after a mean follow-up period of 3.2 ± 1.1 years, the percentage of incident stroke/TIA was significantly higher in the study group than in the control group in the present study. Multivariable Cox regression analysis indicated that moderate/severe LA SEC was an independent predictor of stroke/TIA in the current study. A previous investigation indicated that dense LA SEC was associated with increased thromboembolic risk (16). Gedikli et al. also observed that dense LA smoke showed a significant association with late stroke/TIA events (OR = 3.72) (5).

### SEC and DRT

DRT is one of the most concerning events after LAAC. In the PROTECT-AF study, the incidences of DRT were 1.4, 3.9, and 2.5% at 45 days, 6 months and 1 year after the procedure, respectively (17). Fauchier et al. reported that post-procedural anticoagulation and DAPT were protective factors against DRT, indicating that anticoagulation and antiplatelet therapy are important (18). Sedaghat et al. reported that patients with DRT after LAAC had higher degrees of SEC grades within the LAA (7). The incidence of DRT was higher in the study group than in the control group in our investigation. Three patients with DRT were detected 6 weeks after the procedure when taking warfarin, and the remaining 4 were detected at 6 months and 12 months after the procedure when taking aspirin and/or clopidogrel. This finding suggests that (1) endothelialization in some patients may require more time; (2) LA SEC may increase the rate of DRT before complete endothelialization; (3) NOACs may be better than warfarin within 6 weeks after LAAC; and (4) the combination of aspirin and/or clopidogrel after 6 weeks may be ineffective in preventing DRT in some patients.

### DRT and Stroke

In the present study, moderate/severe LA SEC was associated with a higher risk of DRT and stroke/TIA after LAAC. However, none of the patients with DRT experienced stroke/TIA during the follow-up period. In a multicenter investigation regarding LAAC using an ACP device, DRT was also not associated with an increased risk for thromboembolic events (19). However, Fauchier et al. reported that DRT was an independent predictor of ischemic stroke and TIA during follow-up (HR = 4.39; 95% CI: 1.05–18.43; *P* = 0.04) (18). The real incidence of DRT after LAAC may have been underestimated in most studies, considering the number of patients who did not undergo LAA imaging during follow-up. Although the prognosis associated with DRT is poorly known, we should pay more attention to increasing the frequency of LAA imaging follow-up to evaluate the condition of the LAAC device.

### Antithrombotic Strategy After LAAC

The medication and duration of antithrombotic therapy after LAAC are controversial in the real clinical setting. Under ideal conditions, antithrombotic medication should be used until the presence of complete endothelialization on the atrial surface of the LAAC device. An animal study showed that endothelial cells covered the endocardial surface with smooth muscle cells by 45 days, and complete endothelialization was finished by 90 days after Watchman device implantation (20). In another canine study, by 90 days, there was complete coverage of the ACP atrial surface by stable mature neointima, with diffuse ingrowth of mature fibrous connective tissue in the device and within the surface neointima (21). These preclinical studies provide some guidance on the duration of antithrombotic therapy prior to complete device endothelialization. However, delayed endothelialization often occurs in some patients (22, 23), suggesting that device implantation in humans may require more time to finish endothelialization than that shown in preclinical studies. Therefore, the medication and duration of antithrombotic medication after LAAC, especially in patients with high stroke risk (e.g., with LA SEC), should be determined based on the following conditions: (1) stroke and bleeding risks; (2) adherence to the prescribed regimen; and (3) the process of endothelialization based on TEE and CT follow-up. The majority of patients received OAC at discharge and were transferred to DAPT after 6 weeks, but the incidence of bleeding events was low. Therefore, OAC or DAPT was found to be safe for patients after LAAC. TEE or CT follow-up should be finished at the scheduled time point to evaluate the process of endothelialization and therewith determine the duration of the OAC or DAPT regimen.

The stroke risk was higher in patients with moderate/severe LA SEC. Most late strokes occurred 2.5 years after LAAC, which indicated that long term SAPT for patients with LA SEC may be not sufficient. The antithrombotic regimen was chosen decided according to the previous study (6). However, the antithrombotic regimen after LAAC was still controversial, especially in some subset patients (e.g., patients with prior stroke/bleeding, and with LA SEC, et al.). There was no data comparing different antithrombotic regimens after LAAC in patients with LA SEC. Therefore, randomized controlled trail is needed to answer this question.

### Clinical Implications

As mentioned above, the incidence of DRT and stroke was higher in patients with moderate/severe LA SEC. Did it mean that moderate/severe should be a contraindication for LAAC? The stroke and bleeding risks in the included patients in the present study were both relatively high. The reduction of annual stroke risk was 51%. However, none of the patients suffered major bleeding periprocedure or during follow-up. Therefore, this subset of patients may also benefit from LAAC by significantly reducing the risk of bleeding and moderately decreasing the risk of stroke.

LA SEC was a pre-thrombotic status. In our previous study, we found that LAA thrombus/SEC was associated with a higher percentage of prevalent stroke (OR = 5.13). The LAA voltage was lower in patients with LAA thrombus/SEC. LAA voltage had a negative relationship with LA size in that study (24). The findings suggested that fibrotic remodeling of the atrial substrate may be more serious in this subset of AF patients. Atrial fibrosis was demonstrated to be independently associated with this echocardiographic finding and clinical stroke. Replacement of healthy atrial tissue with fibrosis presumably leads to compromised contractility of the LA and consequent blood stasis (15). Bulic et al. (25) reported a case of extra-appendage left atrial thrombus after LAAC. A large intracardiac, echogenic mass within the LA was detected in a patient who underwent LAAC using Amulet Occluder 4 years ago for persisting LAA thrombus under OAC. The patient was further scheduled to undergo cardiac magnetic resonance imaging and suggested the presence of LA fibrosis as a potential predisposing factor for LA thrombus. This subset of patients (with prior LA thrombus or LA SEC) may be at high risk of both LAA and extra-appendage thrombus. Therefore, adequate evaluation of LA function (e.g., LA strain or fibrosis) should be performed before the procedure in patients with dense LA SEC. Patients with LA SEC who are willing to undergo LAAC should be informed that the risk of stroke may be higher than those without LA SEC.

### Study Limitations

There were several limitations: (1) This was a single-center investigation. However, the study sample was large, and all the procedures were performed by the only master hand (HMC) in our center. Thus, the bias caused by different operators could be neglected. (2) Approximately 82% of patients finished TEE or CT examination at least once during follow-up. Thus, the rate of DRT may have been underestimated. However, the follow-up rate was similar to that in previous studies. (3) Finally, we did not analyze whether the incident strokes were likely to be embolic or related to vascular disease. However, two patients experienced stroke in the control group, and 1 in the study group had carotid plaque at baseline. A total of 9 patients received intracranial CT angiography, and only 1 had plaque. Therefore, the majority of strokes in the present study were more likely to be embolic.

## Conclusions

LA SEC is commonly detected by TEE in patients undergoing LAAC. LA SEC did not increase the periprocedural stroke risk in this study but was associated with a higher risk of DRT. Multivariable analysis indicated that moderate/severe LA SEC was an independent predictor of stroke/TIA during a follow-up period of 3.2 ± 1.1 years. However, DRT was not associated with an increased risk for stroke/TIA. Routine imaging follow-up should be arranged for this subset of patients to evaluate the process of endothelialization, and individual antithrombotic therapy should be determined based on the results of the examination.

## Data Availability Statement

The raw data supporting the conclusions of this article will be made available by the authors, without undue reservation.

## Ethics Statement

Written informed consent was obtained from the individual(s) for the publication of any potentially identifiable images or data included in this article.

## Author Contributions

BW, ZW, and GF designed the study and drafted the manuscript. BW, HW, and WZ collected patient data and edited the images. BW and ZW did the statistical analysis. SZ, BH, and HC critically revised the manuscript and approved the article. All authors contributed to the article and approved the submitted version.

## Funding

This study was funded by the Basic Public Welfare Research Project of Zhejiang Province (Grant Number LGJ20H20001).

## Conflict of Interest

The authors declare that the research was conducted in the absence of any commercial or financial relationships that could be construed as a potential conflict of interest.

## Publisher's Note

All claims expressed in this article are solely those of the authors and do not necessarily represent those of their affiliated organizations, or those of the publisher, the editors and the reviewers. Any product that may be evaluated in this article, or claim that may be made by its manufacturer, is not guaranteed or endorsed by the publisher.
