# Supplementary material for: Left Atrial Spontaneous Echo Contrast and Ischemic Stroke in Patients Undergoing Percutaneous Left Atrial Appendage Closure
Source: Front Cardiovasc Med. 2021 Sep 23;8:723280. doi: 10.3389/fcvm.2021.723280 (PMC8495018; doi:10.3389/fcvm.2021.723280)
Supplement: Supplementary file 1 [file Data_Sheet_1.docx]

Supplementary Material

**Supplementary Table 1** Baseline characteristics of the study population according to device

|  | **Nitinol Cage (n=253)** | **Nitinol Plug (n=155)** | **P value** |
| --- | --- | --- | --- |
| Age, years | 69.5±8.6 | 69.5±9.0 | 0.980 |
| Male | 162 (64.0) | 100 (64.5) | 0.921 |
| Body mass index, kg/m^2^ | 24.5±3.6 | 24.2±3.2 | 0.365 |
| Paroxysmal atrial fibrillation | 89 (35.2) | 43 (27.7) | 0.031 |
| Hypertension | 174 (68.8) | 100 (64.5) | 0.374 |
| Coronary artery disease | 26 (10.3) | 12 (7.7) | 0.393 |
| Diabetes | 44 (17.4) | 27 (17.4) | 0.994 |
| Congestive heart failure | 31 (12.3) | 20 (12.9) | 0.847 |
| Previous stroke/TIA | 189 (74.7) | 109 (70.3) | 0.333 |
| Previous bleeding | 59 (23.3) | 49 (31.6) | 0.065 |
| CHA_2_DS_2_-VASc score | 4.6±1.5 | 4.5±1.5 | 0.498 |
| HAS-BLED score | 3.2±1.0 | 3.1±0.9 | 0.533 |
| Left atrial diameter, mm | 44.7±7.6 | 45.3±7.0 | 0.443 |
| LVEF, % | 62.1±6.6 | 61.5±6.7 | 0.343 |
| LAA orifice diameter, mm | 23.4±3.9 | 27.0±5.4 | <0.001 |
| LAA depth, mm | 29.6±5.2 | 29.5±6.6 | 0.836 |
| Moderate/severe LA SEC | 23 (9.1) | 18 (11.6) | 0.411 |

LAA = left atrial appendage; LVEF = left ventricular ejection fraction; SEC = spontaneous echo contrast; TIA = transient ischemic attack.

**Supplemental Table 2** Follow-up data according to device

|  | **Nitinol Cage (n=253)** | **Nitinol Plug (n=155)** | **P value** |
| --- | --- | --- | --- |
| Adverse events |  |  |  |
| Death | 0 | 1 (0.6) | 0.380 |
| Stroke/TIA | 7 (2.8) | 5 (3.2) | 0.772 |
| Cerebral hemorrhage | 0 | 1 (0.6) | 0.380 |
| Gastrointestinal bleeding | 1 (0.4) | 0 | 1.000 |
| TEE or CT follow-up | 208 (82.2) | 127 (81.9) | 1.000 |
| Imagine follow-up results |  |  |  |
| DRT in patients with TEE/CT | 4 (1.9) | 3 (2.4) | 1.000 |
| Residual flow>5mm | 0 | 0 | 1.000 |
| Residual flow≤5mm | 20 (7.9) | 12 (7.7) |  |

DRT = device-related thrombus; TIA = transient ischemic attack; TEE = transesophageal echocardiography.

**Supplementary Table 3** The incidence of DRT and stroke/TIA in patients with OACs vs. DAPT after LAAC

|  | **OACs (n=377)** | **DAPT (n=31)** | **P value** |
| --- | --- | --- | --- |
| CHA_2_DS_2_-VASc score | 4.6±1.5 | 5.1±1.5 | 0.054 |
| HAS-BLED score | 3.1±0.9 | 3.5±0.7 | 0.027 |
| LA SEC |  |  | 0.360 |
| None | 275 (72.9) | 24 (77.4) |  |
| Mild | 43 (11.4) | 2 (6.5) |  |
| Mild to moderate | 23 (6.1) | 0 |  |
| Moderate | 25 (6.6) | 4 (12.9) |  |
| Severe | 11 (2.9) | 1 (3.2) |  |
| TEE/CT follow-up | 310 (82.2) | 25(80.6) | 0.825 |
| DRT | 6 (1.9) | 1 (4.0) | 0.422 |
| Stroke/TIA | 11 (2.9) | 1 (3.2) | 1.000 |

APT = antiplatelet therapy; DRT = device-related thrombus; LA = left atrium; LAAC = left atrial appendage closure; OAC = oral anticoagulation; SEC = Spontaneous echo contrast; TEE = transesophageal echocardiography; TIA = transient ischemic attack
